# Exploring clinical empathy among maternal healthcare providers in Zambia: Does the heart meet the mind? Insights from a qualitative study

**DOI:** 10.1177/13591053251378961

**Published:** 2025-10-15

**Authors:** Lena Halawi, Francis Sichimba, Kalunga Cindy Nakazwe, Atika Khalaf

**Affiliations:** 1 Kristianstad University, Kristianstad, Sweden; 2 University of Zambia, Lusaka, Zambia; 3 Mohammed Bin Rashid University of Medicine and Health Sciences, Dubai Health, Dubai, United Arab Emirates

**Keywords:** clinical empathy, detached concern, maternal healthcare, emotional engagement, clinical objectivity

## Abstract

Empathy is crucial for enhancing interpersonal interactions in healthcare. While provider empathy improves health outcomes, studies focused on this concept in Zambia are limited. This qualitative study utilized a hermeneutic phenomenological approach, employing qualitative content analysis as the analysis method, to explore clinical empathy through the perspectives of 14 maternal healthcare providers recruited via purposive and snowball sampling. Participants varied in age, experience, and professional roles. The study identified three primary themes: (1) the multifaceted nature of empathy in maternal healthcare, (2) dual aspects of empathy—patient care and professional boundaries, and (3) contextual dynamics—balancing challenges in maternal empathy. Findings highlight that while empathy enhances patient experiences, providers often struggle to maintain emotional boundaries. The study highlights the need for targeted training programs in strengthening empathy in clinical practice and recommends further research on culturally specific expressions of empathy in healthcare settings.

## Introduction

Empathy is vital for improving interpersonal interactions in clinical healthcare, promoting healing beyond medical treatments (Decety and Fotopoulou, 2015; Reynolds and Scott, 2000). However, a lack of a unified theoretical framework for empathy leads to confusion and oversimplifies it into cognitive and affective dichotomies (Guidi and Traversa, 2021; Halpern, 2011; Shapiro, 2011). This focus on differentiating empathy types skews medical culture toward cognitive empathy, fostering detached concern over genuine emotional engagement (Guidi and Traversa, 2021; Halpern, 2011). Consequently, it limits a comprehensive understanding of empathy in practice (Halpern, 2003, 2011). Guidi and Traversa (2021) note that an emphasis on cognitive empathy may lead to detachment, viewing emotional engagement as a hindrance to objectivity. Halpern (2003, 2011) and Coulehan (2005) warn against prioritizing cognitive precision over emotional connection, advocating for a view of empathy that integrates both dimensions to enrich the healthcare experience and enhance understanding of patient needs.

Empirical evidence indicates that empathy is crucial for effective clinical practice. Research shows that empathic interactions enhance diagnostic accuracy, build patient trust, and improve treatment outcomes (Charitou et al., 2019). Hojat et al. (2011, 2013) highlight that empathy strengthens rapport and fosters deeper patient engagement and collaborative decision-making. Factors like empathy influence healthcare providers’ attitudes (Isangula et al., 2022) and are essential for creating meaningful patient connections and holistic emotional engagement (Irarrázaval and Kalawski, 2022). In maternal healthcare (MHC), empathy helps reduce adverse outcomes by ensuring comprehensive monitoring of both physical and mental health (World Health Organization (WHO), 2024). Recent studies emphasize the importance of empathic communication between maternal healthcare providers (MHCPs) and patients for mothers’ well-being (Aktas and Pasinlioğlu, 2021; Smith et al., 2022). Although interventions aimed at individual clinicians have shown promise in enhancing empathy, there remains a significant gap in organizational-level initiatives (Nembhard et al., 2023).

The consistent application of empathy in healthcare is often lacking, particularly in culturally diverse regions like Africa, Asia, Latin America, and the Middle East. Studies show a decline in empathic interactions among providers, with negative attitudes undermining care (Charles et al., 2024; Mannava et al., 2015). In Zambia, factors such as perceived care quality, social norms, and economic barriers influence MHC service utilization (Sialubanje et al., 2014). Women’s service utilization intentions correlate positively with favorable attitudes, education, and income, while negatively with social norms, age, and distance (Sialubanje et al., 2014). High poverty levels, cultural beliefs, and low education in Zambia affect healthcare interactions (Mangimela-Mulundano et al., 2022). HCPs, respected within the social hierarchy, may hinder patient communication as patients hesitate to express concerns. Additionally, this hierarchy can limit providers’ emotional responsiveness, compromising care quality and support. Broad societal factors influence empathy capacity in healthcare providers (Vanstone and Grierson, 2022). These dynamics underscore the complexities of patient-provider interactions in Zambia and the need for a nuanced understanding of the healthcare experience in this context.

While evidence underscores the importance of empathy in healthcare, there is a notable gap in understanding its complexities in non-Western contexts, particularly in Zambia. Most existing literature focuses on Western settings (Chopik et al., 2017), overlooking diverse cultural interpretations of empathy. Halpern (2014) emphasizes the necessity of contextualizing empathy, as cultural factors significantly shape how healthcare professionals express and perceive empathic interactions. Given the critical role of empathy in MHC, it is vital to explore how MHCPs in Zambia interpret and practice empathy within local dynamics. Research indicates that empathic interactions directly influence patient outcomes (Agbi et al., 2023; Nembhard et al., 2023), necessitating a patient-centered approach that improves MHC utilization and clinical outcomes. Clinical empathy research must consider provider perspectives, contextual factors, and key barriers that influence healthcare providers’ attitudes toward empathy. Furthermore, there is a notable research gap concerning organizational-level interventions aimed at enhancing empathy in care delivery. This study, therefore, aims to identify the unique contextual factors impacting the practice of empathy in Zambia’s MHC services.

Clinical empathy can be rigorously conceptualized within the African cultural context by integrating both traditional and contemporary models of empathy while recognizing the unique cultural nuances that shape interpersonal interactions in healthcare. African cultures often emphasize communal values, respect, and emotional interconnectedness (Ajitoni, 2024), contrasting with Western individualistic approaches. While neurobiological and psychosocial models of empathy focus on cognitive recognition and emotional engagement (Guidi and Traversa, 2021), they may neglect the cultural context’s role in shaping empathetic responses. The authors argue that non-verbal communication, storytelling, and shared experiences enhance empathic understanding relationally, rather than purely cognitively. Additionally, the focus on collective well-being in many African societies necessitates a model of empathy that incorporates moral and ethical dimensions, highlighting the provider’s role in communal health. By recognizing these aspects, a more holistic and culturally relevant framework for clinical empathy can promote genuine engagement and healing for both patients and healthcare providers.

This study explores empathy in Zambia’s MHC system, aligning with Aaron Antonovsky’s salutogenic model, which views health experiences as comprehensible, manageable, and meaningful (Antonovsky, 1979, 2005). By examining how MHCPs express and practice empathy, the research highlights the cultural and contextual factors that significantly influence patient outcomes.

Furthermore, Halpern’s (2011) concept of clinical empathy, defined as “engaged curiosity,” provides a valuable framework for understanding MHC in Zambia. It highlights the proactive role of healthcare providers in comprehending patients’ experiences, fostering deeper connections beyond mere sympathy. This approach is crucial for addressing the emotional and psychological needs of mothers during the perinatal period, empowering them and enhancing their resilience amid healthcare challenges. The literature indicates a decline in empathetic interactions among providers, suggesting that an emphasis on cognitive precision may hinder emotional understanding. Therefore, this study underscores the importance of cultural sensitivity and the need for MHCPs to cultivate empathic relationships rooted in curiosity to support mothers effectively.

On the other hand, Antonovsky’s salutogenesis emphasizes the origins of health, focusing on factors that promote well-being rather than just addressing illness. This shifts the narrative toward identifying and nurturing the strengths within individuals and communities. When paired with Halpern’s engaged curiosity, a holistic understanding of maternal health emerges. Together, they highlight the importance of empathic engagement and addressing the emotional and psychological needs of mothers during the perinatal period, fostering effective, patient-centered healthcare practices that integrate empathy and cultural understanding.

Moreover, the prior research highlights concern about the decline of empathic interactions among healthcare providers, suggesting that an emphasis on cognitive precision may hinder emotional understanding and affect maternal well-being. This study integrates empathy with Antonovsky’s salutogenesis and Halpern’s engaged curiosity to explore how cultural nuances shape empathic practices among MHCPs in Zambia. By examining the beliefs and values that influence empathy, it aims to improve MHC outcomes and address the existing literature gap. This research provides insights into the definitions of empathy and the factors affecting empathic interactions, enriching the discourse on MHC and informing strategies for improved patient outcomes tailored to the unique needs of women in Zambia.

## Method

### Study design

This study employed a qualitative approach, utilizing a hermeneutical phenomenological design complemented by content analysis. The hermeneutical phenomenological design was chosen for its suitability in capturing the complex and nuanced nature of empathy among MHCPs in Zambia. This design integrates the principles of phenomenology, which focuses on describing perceived lived experiences, with hermeneutics, which focuses on the role of interpretation, context, and history in understanding a phenomenon (Fuster, 2019). This integration offers a comprehensive view of human experiences, making it particularly relevant for exploring empathy among MHCPs in Zambia. Consequently, our selection of methods reflects a hermeneutical phenomenological approach. This is evident in our focus on gathering empirical data regarding the MHCPs’ experiences of clinical empathy, alongside the analytical techniques used to interpret the meanings underlying those experiences.

Furthermore, the study was guided by an overarching aim rather than specific hypotheses or research questions, in line with qualitative research conventions.

### Context and participants

This study examines MHC in Lusaka, Zambia, highlighting the significant challenges within its healthcare system, including limited resources, high patient-provider ratios, and inadequate infrastructure (Clarke-Deelder et al., 2022; Jacobs et al., 2023). Despite these issues, Lusaka’s healthcare infrastructure is comparatively better than in rural areas, offering greater access to specialized care (Khondowe and Mpundu, 2024). MHCPs in Lusaka face demanding work conditions, often in overcrowded facilities with insufficient resources (Nankamba and Mwanaumo, 2024; Zimba et al., 2022). The socio-economic and cultural context, including traditional practices and gender roles, significantly influences patient care and provider relationships (Ngulube et al., 2024). This context underscores the importance of understanding the experiences and perceptions of empathy among MHCPs, as it can illuminate how empathy is woven into clinical practice and its effects on patient outcomes.

This study included 14 MHCPs from various healthcare facilities in Lusaka, comprising 11 public and 3 private institutions, with a diverse range of experiences in maternity care. Participants, including six nurses, seven clinical officers, and one medical practitioner aged 20–39, contributed valuable insights into the integration of empathy in maternity clinical practice. Understanding these dynamics is crucial for improving healthcare delivery and patient-provider relationships in Lusaka.

### Data collection

The 14 interviews were conducted between January 2024 and April 2024 by the second and third authors (FS, KCN) to ensure consistency and reliability in the data collection process. Prior to interviews, the MHCPs received full information about the study, including its objectives, methods, and potential benefits and risks. They were assured of anonymity and confidentiality to encourage open and honest responses. Informed written and verbal consent was obtained from each participant before proceeding with the interviews. The two researchers involved in data collection are psychologists who understand the importance of empathy in interactions. In recognition of potential biases that are inherent in qualitative data collection, the researchers maintained a reflective journal. The researchers bracketed their biases and assumptions to ensure an objective and open understanding of the participants’ views and experiences. Hence, reflexivity was employed throughout the data collection, analysis, and writing phase to ensure the trustworthiness of the findings.

A self-developed, semi-structured interview guide was designed by the research team to explore the providers’ understanding, perceptions, and experiences of empathy in the clinical context. The interview guide included a set of exemplary questions aimed at eliciting comprehensive and nuanced responses. Examples of these questions were: *“How would you define empathy in the context of patient care?,”* and *“In your experience, how challenging is it to express empathy when providing care to patients?*” To facilitate fruitful discussion and attain rich, detailed content, supplementary questions in the form of probes were used whenever necessary. These probes helped to clarify and expand on the participants’ responses, ensuring that comprehensive answers were obtained. Each interview lasted 30–60 minutes, allowing for an in-depth exploration of the participants’ experiences and perceptions without causing undue fatigue or discomfort.

### Data analysis

Qualitative content analysis, following Graneheim and Lundman’s (2004) framework, was used to analyze interview transcripts, considering both manifest (obvious meaning) and latent (underlying meaning) content. An inductive approach was employed to extract these contents. The coding process was manual, beginning with a careful review of transcripts to identify meaning-bearing units, that is, groups of words that express a core idea (Graneheim and Lundman, 2004). Abstraction followed, leading to the condensation and coding of units while preserving their essential components. This part of the analysis involved the use of codes, categories, and themes. Codes were assigned to specific meaning units, contextualizing objects and events. These codes were then organized into categories, grouping content that shared characteristics and represented the manifest content (Graneheim and Lundman, 2004). Finally, patterns among categories were identified to explore the deeper latent meanings, culminating in themes.

While the coding framework and its justification, as well as the primary theme identification for this study, were executed by a single researcher, the first author (LH), the assessment of the identified themes by all co-authors was instrumental in upholding the reliability of the findings. Each author engaged in a thorough review of the core themes and codes, sharing their unique viewpoints and insights. This collective approach invited a variety of perspectives and critical dialog, strengthened the credibility of our conclusions, reduced the risks associated with bias from a single coder, and enhanced the inter-coder reliability. To strengthen the integrity of the analysis, the main themes were cross-checked collectively against the original data and pertinent citations from the interviewed MHCPs. This final step was essential for ensuring the trustworthiness and validity of the findings, reflecting commitment to a thorough and ethical research process.

### Ethical considerations

This study was approved by the University of Zambia, School of Humanities Institutional Review Board (IRB; Ref IORG No. 0005376) on November 28th, 2023. The research ethics adhered to the principles outlined in the Declaration of Helsinki. Informed and verbal consent were obtained from all participants before the interviews.

## Results

The inductive qualitative content analysis brought forth three main themes: (1) *The Multifaceted Nature of Empathy in Maternal Healthcare – From Conceptual Understanding to Practical Application* (2) *The Dual Nature of Empathy in Maternal Healthcare – Enhancing Patient Care while Navigating Professional Boundaries and* (3) *Contextual Dynamics of Empathy in Maternal Healthcare – Balancing Challenges and Cultivating a Patient-centered Approach* (see Supplemental Material 1). In addition, the themes and categories have been aligned with different dimensions of clinical empathy as perceived by the participants (see Supplemental Material 2).

### The multifaceted nature of empathy in maternal healthcare – From conceptual understanding to practical application

The MHCPs’ narratives showed a nuanced understanding of clinical empathy within the context of maternal healthcare, encapsulated in the first theme, *“The Multifaceted Nature of Empathy in Maternal Healthcare - From Conceptual Understanding to Practical Application”*. This theme emerged from two distinct categories: *“Holistic Emotional Engagement in Patient-Centered Care – The meaning and attributes of empathy”* and *“Not Practical Enough” – The Disconnect Between Theoretical Knowledge and Practical Application of Clinical Empathy.*

#### “Holistic emotional engagement in patient – centered care- The meaning and attributes of empathy”

The MHCPs agreed on the significance of empathy as both a characteristic of a caregiver and an actionable element in patient interactions. This was evidenced in their conceptualization of empathy, which combined cognitive empathy that is, understanding a patient’s circumstances, with affective empathy that is, emotionally engaging with patients. One MHCP articulated that sometimes, empathy is shown in that *“you don’t really have to show it.”* Expanding on this point, the respondent said: *“Mostly, I would be really attentive and let them talk, not interrupting them. Keep nodding. Give them undivided attention,”* emphasizing that non-verbal cues such as attentive listening, nodding, and providing undivided attention can effectively convey understanding. This aligns with the idea that empathic engagement extends beyond mere words; it includes a holistic approach to nurturing the patient-provider relationship.

Despite general consensus on the importance of empathy, there were discriminating opinions concerning emotional involvement. Some MHCPs expressed that emotional commitment might not be necessary for effective empathy. *"You do not need to attach emotion. Not as if you have that problem yourself or are a part of it. Don’t look like you are also a part of it,”* one MHCP stated, suggesting a parsimonious approach to emotional engagement that prioritizes professional boundaries while still fostering an empathic connection. This highlights a critical consideration in the application of empathy; it is not solely about emotional involvement, but also about recognizing the diverse interpretations and expressions of empathy across different healthcare contexts.

#### “Not practical enough” – The disconnect between theoretical knowledge and practical application of clinical empathy

While the importance of empathy was widely acknowledged, respondents noted that its application in clinical practice often fell short of theoretical ideals. Some MHCPs expressed concern over the practicality of implementing empathetic behaviors, particularly under the constraints of time and cultural factors that could affect the patient-provider relationship. As one MHCP reflected, establishing a connection requires patience and kindness: *“If you are not empathetic, you will not be patient and kind in explaining and trying to make the client understand. This is why empathy is key.”* This speaks to the belief that empathy is a foundational quality, necessary for effective consultations, one that profoundly influences both the provider’s demeanor and the patient’s comfort level during vulnerable moments.

The MHCPs also highlighted the significance of understanding patients’ unique emotional states, especially for new mothers undergoing significant physiological and psychological changes. The narratives emphasized the role of empathy in uplifting patients during their medical journeys, reinforcing the sentiment that *“empathy comes in very handy”* in delicately navigating individual patient circumstances in maternity care. Adding to this perspective, the MHCP said, *“You have to realize that the period of pregnancy is quite a delicate period. It is subjective. So, every client that you meet is different from a previous client. So, empathy comes in handy because you really have to understand the divisions.”* Such an insight underscores the need for healthcare professionals to adopt a patient-centered approach that acknowledges and addresses specific needs, thereby facilitating better communication and enhancing overall care quality.

The MHCPs noted that cultural backgrounds significantly shape the patient-provider dynamic, highlighting the importance of recognizing patients’ diverse experiences to foster open dialog, especially during pregnancy. Empathy emerged as crucial for effective interaction and for conveying complex medical information. It transcends cognitive understanding, becoming integral to daily interactions with maternity patients. These findings underscore empathy as a multifaceted concept in MHC, emphasizing the need for healthcare providers to combine theoretical knowledge with practical application to address the emotional and medical needs of their patients.

### The dual nature of empathy in maternal healthcare – Enhancing patient care while navigating professional boundaries

The second theme, depicts empathy in MHC as multi-faceted, highlighting its dual role as both a catalyst for improved patient care and a potential challenge to professional boundaries. This theme emerged from two categories: *“Empathy as a Catalyst for Enhanced Healthcare Efficacy and Patient Engagement”* and *“The Empathy-Professionalism Balance: Navigating Emotional Engagement and Clinical Objectivity.”*

#### “Empathy as a catalyst for enhanced healthcare efficacy and patient engagement”

The MHCPs consistently emphasized the significance of empathetic expression in fostering positive clinical outcomes. The providers observed that empathetic interactions with patients allowed for deeper connections, facilitating more comprehensive communication about medical concerns. One MHCP articulated this point effectively: *“Empathy is very important. If you don’t practice it, you chase away the people who come to be served by you. Because of how I relate to them or how I make them comfortable, they will open up and talk about a lot with me, and that does not only help me provide the best service, but it also helps me come up with the best diagnosis and offer the best service. They will not leave any stone unturned. It will make me make an informed decision.”* This highlights not only the necessity of empathy in garnering patient trust but also its reciprocal nature; as patients feel understood and valued, they are more inclined to share their experiences, ultimately leading to improved diagnostic accuracy and care.

Moreover, the emotional dimensions of patient interactions were deemed essential in ensuring safety and trust. The respondents noted that when patients perceive their providers as empathic, they are more willing to disclose vital information and adhere to treatment recommendations. This aligns with the experiences shared by a MHCP working with a mother of a premature baby who shared: *“We made her understand, we carried her weight as well. . . That relationship we built really helped.”* Such connections demonstrate the profound impact empathic engagements can have not only on diagnostic and clinical outcomes but also on the emotional wellness of patients and their families.

#### “The empathy-professionalism balance: Navigating emotional engagement and clinical objectivity”

While the advantages of empathy in enhancing patient care were clear, some healthcare providers raised concerns regarding the potential risks to professional objectivity. Several respondents cautioned that excessive emotional engagement could compromise clinical judgment, leading to emotional overidentification with patients. One MHCP highlighted this risk by stating, *“Yes. With these patients we need to leave a limit. I have got an experience with that. A colleague of mine started mourning with the patients, which was not okay.”* This statement underscores the precarious balance healthcare providers must maintain between showing empathy and retaining professional boundaries.

The recognition that empathy can lead to a therapeutic environment while simultaneously necessitating emotional restraint illustrates the complexity of patient-provider relationships in MHC. Some respondents articulated that while emotional connection enriches the therapeutic alliance, it is vital to establish boundaries to allow healthcare providers to deliver care effectively without becoming overwhelmed by their emotional responses. Reflecting on this duality, one healthcare provider stated, *“Empathy is very important because it brings out certain issues that the patient is going through, which would help you make certain decisions or how you can handle the patient. . .it becomes easier to provide services to them (patients).”* This highlights that although empathy is valuable for eliciting information and understanding patient issues, it must be practiced judiciously.

The narratives reflect the dual nature of empathy in MHC, elaborating on the intricate balance between enhancing patient care and maintaining professional boundaries. The insights shared by the MHCPs revealed a nuanced understanding of how empathy fosters clinically effective relationships, while also posing challenges to objectivity. Therefore, it becomes essential to cultivate an empathic approach that respects personal and professional limits, ensuring optimal outcomes for both patients and healthcare practitioners.

### Contextual dynamics of empathy in maternal healthcare – Balancing challenges and cultivating a patient-centered approach

The third and final theme, reveals a complex interplay of challenges and facilitators that shape the expression of empathy among male MHCPs. These dynamics are vital in understanding how empathetic care can enhance patient experiences while navigating potential barriers inherent in interpersonal relationships. The third theme emerged from seven categories: *“Navigating Gender-Related Misinterpretations,” “Balancing Professionalism and Patient Familiarity,” “Age and Educational Background as Barriers to Empathy,” “Socioeconomic Factors and Perceptions of Empathy,” “The Role of Personal Beliefs and Emotional Resistance,” “Motivation and Positive Outcomes of Empathy”; and “Environmental Influences on Empathy in Maternal Healthcare.”*

#### “Navigating gender-related misinterpretations”

A significant challenge identified was the gender-related misinterpretations of empathetic gestures. Male MHCPs reported that expressions of empathy could be misconstrued as flirtation, particularly with female patients. This dynamic complicates the professional relationship and may cause anxiety for both parties involved. As one MHCP noted, *“Acting empathetically can sometimes lead patients to become overly familiar, which can blur professional boundaries.”* This illustrates the precarious nature of empathy in a healthcare setting, where intentions may be misinterpreted, causing discomfort and reluctance in MHCPs to fully engage emotionally.

#### “Balancing professionalism and patient familiarity”

Other significant challenges identified were boundary challenges that arise when patients exhibit heightened appreciation for the MHCP’s empathetic gestures, sometimes leading to unwarranted requests for personal contact. This situation highlights the delicate balance MHCPs must maintain between offering compassionate care and upholding professional boundaries. The desire for familiarity can become a double-edged sword, where patients expect ongoing access to their MHCP, complicating the provider’s ability to maintain objectivity. As emphasized by a MHCP’s experience: *“The worst part is when you give them your number. . . At any particular moment, they want to call, they want to consult,”* reflecting a frustrated acknowledgment of how empathy can inadvertently lead to breaches in professionalism.

#### “Age and educational background as barriers to empathy”

Demographic factors such as age and educational background of patients also emerged as relevant challenges. Male MHCPs noted difficulties in connecting with younger female patients (ages 18–25), who were often perceived as closed off, limiting the scope for emotional rapport. Similarly, well-educated female patients in their mid to late 40s presented particular challenges, as they often entered appointments with pre-diagnosed issues and a desire for validation rather than open dialog. One MHCP’s observation: *“If you try to show empathy to them, it’s like a waste of time. . . All they want from you is to validate what they know,”* underscores the notion that patients may seek affirmation rather than genuine empathetic engagement, thereby stunting the development of meaningful interactions.

#### “Socioeconomic factors and perceptions of empathy”

Socioeconomic status further complicates the landscape of empathetic care. MHCPs noted that patients from lower socioeconomic backgrounds might misinterpret expressions of empathy as an invitation for special treatment, which can create tension in the patient-provider relationship. The challenge of separating genuine concern from perceived exploitation became evident in quotes such as: *“When it comes to economic stratification, you have people who are either middle or lower, so you find that those people who mistake empathy for an opportunity to actually get some handouts and all that. It becomes difficult to separate the two.”* This highlights the balancing act MHCPs face in maintaining empathy while managing diverse patient attitudes, emphasizing the need for tactfulness in conveying genuine intentions to avoid misinterpretation. This complexity requires heightened awareness of both verbal and non-verbal communication strategies to effectively connect provider intent with patient reception.

#### “The role of personal beliefs and emotional resistance”

Emotional resistance from patients can also impede the expression of empathy, especially in cases where the healthcare provider’s personal beliefs conflict with patient behaviors. An experience shared about a drunk pregnant woman illustrated the internal struggle faced by one MHCP: *“It was very difficult for me to show empathy because the anger that I had after spending hours. . .and then she comes in that situation.”* This incident reveals how personal feelings of frustration and moral judgment can act as barriers to empathetic communication, reminding us that empathy is not solely about the patient’s experience but is also influenced by providers’ emotional states.

#### “Motivation and positive outcomes of empathy”

Despite the aforementioned challenges, many MHCPs expressed a strong motivation to empathize with patients, driven by the belief that empathy can lead to improved clinical outcomes. One MHCP articulated that: *“If you express empathy in the best way and get the client’s confidence, it leads to proper adherence, which leads to good outcomes in the pregnancy.”* This sentiment encapsulates the notion that empathetic engagement not only fosters trust but also enhances the likelihood of positive health outcomes, ultimately benefiting both the patient and the healthcare provider.

#### “Environmental influences on empathy in maternal healthcare”

Additionally, the findings revealed a significant interplay between the clinical environment and the expression of empathy among MHCPs. It is evident that aspects of the physical setting, including privacy, ventilation, and lighting, profoundly impact both the provider’s ability to empathize with and the patient’s comfort in expressing vulnerabilities. A recurring sentiment among MHCPs was the acknowledgment of the clinical environment as a critical factor that influences empathic interactions. One MHCP stated succinctly: *“The environment matters most,”* highlighting the overarching importance of a supportive physical setting. This underscores the notion that empathy is not merely a personal attribute of the healthcare provider, but rather a cultivated response facilitated by contextual factors. Several MHCPs pointed out that the lack of privacy, due to shared rooms and inadequate spatial arrangements, creates barriers to open communication. For instance, a MHCP expressed concern over how *“the current working environment lacks privacy,”* indicating that the presence of screens dividing spaces can inhibit patient openness. Such infrastructural shortcomings not only hamper empathic exchanges but can also contribute to an atmosphere of discomfort, which diminishes the quality of care provided. Further emphasizing the need for a conducive atmosphere, another MHCP articulated that their empathetic expression is *“discouraged if the situation does not allow privacy.”* This highlights the critical relationship between the clinical environment and the psychological safety of both patients and providers. The importance of privacy is echoed in their desire for a *“room* that is *more ventilated”* and *“a comforting consultation room,”* which collectively contribute to a therapeutic environment that respects patient confidentiality and fosters trust.

Moreover, the adverse characteristics of clinical settings, such as poor ventilation, inadequate lighting, and noise, hinder effective communication and empathy. Recognizing these barriers highlights that intent alone is not enough; a supportive environment is essential. As one MHCP noted, *“It should have privacy,”* emphasizing the need for healthcare spaces that enhance patient comfort and confidentiality. The insights from MHCPs reveal the importance of balancing current clinical challenges with a patient-centered approach, as empathetic connections can only thrive in environments that prioritize privacy and support.

Empathy in MHC is complex, but understanding interpersonal dynamics can help MHCPs provide compassionate care while maintaining professional boundaries. Insights from their experiences emphasize the need for better training and support to navigate emotional interactions. By addressing these factors, healthcare systems can enhance MHCPs’ ability to express empathy, improving the quality of maternal care.

## Discussion

Concerns about depersonalized and emotionally distant care in healthcare settings are widely recognized for their negative impact on patient trust, satisfaction, and outcomes (Hojat et al., 2023; Shapiro, 2011). This study, the first of its kind in Zambia, explored MHCPs’ perceptions and practices of clinical empathy, offering a culturally embedded lens through which to understand how empathy is enacted, constrained, and interpreted in low-resource maternity care contexts. The findings support the growing consensus that empathy is not a fixed trait, but a dynamic and context-sensitive practice shaped by professional norms, emotional labor, and sociocultural structures.

Our results reinforce the theoretical shift from viewing empathy as purely cognitive or emotional toward a more integrated model. MHCPs described empathy as involving verbal and non-verbal responsiveness, active listening, and emotional attunement, elements that align with Halpern’s (2014) concept of emotionally engaged curiosity, where understanding emerges from emotional resonance, not detached reasoning. This view also echoes Shapiro’s (2011) assertion that cognitive perspective-taking alone is insufficient for genuine empathic engagement. Furthermore, our findings align with Moudatsou et al. (2020), who conceptualize clinical empathy as a multidimensional construct involving cognitive, affective, behavioral, and ethical components.

While MHCPs expressed commitment to empathetic care, recognizing its role in fostering patient trust, improving communication, and enhancing outcomes, they also reported significant challenges in translating these ideals into practice. These included systemic constraints such as time pressure, lack of privacy, and emotional fatigue, as well as interpersonal challenges like gendered misinterpretations and cultural barriers. This reflects findings from other low-resource settings, where empathy is constrained by infrastructural deficits and institutional norms (Isangula et al., 2022; Mammeri et al., 2020).

In contrast to studies from high-income settings that highlight “empathy fatigue” (Lampert and Glaser, 2018), Zambian MHCPs often showed sustained emotional engagement, shaped by communal cultural values and relational care expectations. However, tensions were evident. Some providers reported withholding emotional expression to preserve clinical objectivity, while others hesitated to show empathy to certain patient groups, such as well-educated or younger women, revealing the presence of unconscious biases. These findings suggest the need for training that fosters reflective practice and mitigates bias, particularly in emotionally complex and hierarchical care environments.

Gender dynamics further complicated the practice of empathy. Male providers reported concerns about their intentions being misinterpreted, especially in emotionally intimate maternity care settings, while female MHCPs faced cultural expectations to embody emotional warmth and caregiving, even as they managed professional boundaries. Such dynamics underscore the emotional labor embedded in clinical roles, especially for women, and the need for gender-sensitive support systems in healthcare institutions (Kalindi et al., 2023).

Cultural context also shaped empathic communication. In Zambia, patient–provider interactions are often grounded in communalism, hierarchical respect, and indirect or non-verbal communication styles, such as tone, posture, and gestures, which serve as critical modes of emotional connection (Mubanga-Chilonga et al., 2025; Mwamba et al., 2022). These culturally embedded practices highlight that models of empathy developed in Western contexts may not fully capture how empathy is expressed or interpreted in Sub-Saharan Africa.

The transformative potential of empathy was consistently emphasized by participants, aligning with broader movements toward patient-centered care (Karimi and Abdollahi, 2019; Rozenblum et al., 2015). When enacted effectively, empathy enhances patient openness, fosters relational continuity, and supports better clinical outcomes (Kerasidou et al., 2021). However, organizational barriers, such as high patient-provider ratios, spatial constraints, and systemic underinvestment, can erode empathic capacity and reduce care quality. Literature shows that empathic communication is shaped by interpersonal, institutional, and environmental factors (Terrasi, 2022), reinforcing the need for systemic solutions.

To address these challenges, MHCPs may benefit from strengthening emotional intelligence and reflective capacity, helping them manage the emotional complexity of caregiving while maintaining professional boundaries (Gamage and Sudusinghe, 2024). Supporting empathic practice also requires institutional reform, such as the redesign of clinical spaces and workflow processes, to better accommodate relational care under resource constraints.

The adoption of the WHO’s Integrated People-Centred Health Services (IPCHS) model, which emphasizes care coordination around individuals’ needs, supports the relevance of our findings (WHO, 2016). By promoting responsiveness, communication, and holistic care, the IPCHS framework aligns with the core elements of clinical empathy identified in this study. Its integration into Zambia’s MHC system could institutionalize empathy, address systemic barriers such as fragmented services, and improve continuity of care. Evidence shows that people-centred approaches enhance both patient satisfaction and provider engagement, contributing to more meaningful interactions (WHO, 2016). This reinforces our recommendations for structural reforms and context-specific training to strengthen empathy as a core component of high-quality MHC.

Finally, Antonovsky’s (1987) salutogenic framework offers a useful lens to interpret our findings. His model emphasizes creating environments that foster well-being, rather than merely treating illness, by supporting individuals’ sense of coherence: comprehensibility, manageability, and meaningfulness. MHCPs who engage with patients empathically not only respond to distress but help patients make sense of their experiences, thereby contributing to resilience and emotional recovery. Conversely, systemic barriers and emotional dissonance can undermine this process, reducing both provider well-being and care quality. By cultivating emotional self-awareness and institutional support, healthcare systems can promote a more salutogenic culture, one that prioritizes relational care, patient dignity, and provider resilience.

### Significant implications

This study highlights the nuanced role of empathy in healthcare, emphasizing its value and the risks associated with its mismanagement. Meeting patients’ emotional needs is essential to improving provider-patient relationships (Nembhard et al., 2023). To strengthen maternity care in Zambia, empathy should be integrated into healthcare systems through structured support and formalized communication training (Watts et al., 2023). Clinical education and ongoing professional development must explicitly address context-specific challenges, including gender dynamics, emotional labor, and boundary-setting. Institutional leadership and policymakers should collaborate to embed experiential, culturally sensitive empathy training within health education programs (Van Den Bossche and Baktiran, 2021). Supportive environments that encourage reflective practice and open discussion of empathic care can reduce barriers and promote emotional resilience. Reforming medical curricula to emphasize emotional intelligence, potentially through the inclusion of humanities and social science perspectives, can foster long-term change (Watts et al., 2023). Future research should explore practical strategies to implement and evaluate empathy training while balancing emotional attunement with professional boundaries. The study offers a foundation for developing targeted interventions to enhance empathy and improve the quality of maternity care in Zambia. Developing structured, culturally adapted training modules that include role-playing, reflective practice, and emotional intelligence development would be a meaningful step forward.

### Methodological limitations

This study provides qualitative insights into the perceptions of clinical empathy among MHCPs in Zambia, but it has several limitations. First, despite employing multiple coders and standardized procedures to enhance credibility (Graneheim et al., 2017), researcher influence could affect theme generation and data categorization, especially given the hierarchical context of the interviews. Two members of the research team were from the same cultural context as the participants, which may have enhanced contextual understanding but also introduced potential for insider bias. To mitigate this, the primary analysis was conducted by the first author, who did not share this cultural background and thus brought a degree of analytical distance. While this approach reduced cultural preconceptions, it may also have limited sensitivity to certain culturally embedded meanings (Berger, 2013). Nonetheless, a reflexive and collaborative triangulation process helped ensure that the final themes remained grounded in participants’ narratives and accurately reflected the sociocultural context (Finlay, 2002; Patton, 2015).

Cultural bias complicates the findings, as participants’ interpretations of empathy are shaped by local norms and communication styles, limiting generalizability to other settings, particularly outside Sub-Saharan Africa or in more advanced health systems. Additionally, the small sample size from public healthcare facilities restricts perspective diversity, and the absence of patient voices or observational data could lead to *social desirability bias*, skewing self-reported attitudes.

While the inductive content analysis enabled rich exploration of MHCPs’ experiences, it may have underemphasized variation across subgroups. Although the sample included gender and role diversity, it was not intended to be statistically representative. As in all qualitative research, the goal is not generalizability but transferability to similar contexts, guided by data saturation and purposive sampling (Lincoln and Guba, 1985; Patton, 2015). The absence of patient perspectives and limited rural representation may constrain broader applicability. Nonetheless, we ensured trustworthiness through an audit trail, transparent coding, and reflexive dialog (Elo et al., 2014; Nowell et al., 2017).

## Conclusion

This study explores how MHCPs in Zambia view and practice clinical empathy in maternity care. It finds that while empathy is essential for patient trust and communication, its implementation faces challenges such as high patient volumes, gender misinterpretations, lack of privacy, and balancing emotional involvement with professional boundaries. The findings highlight the need for a structural approach to enhancing empathy in clinical settings.

The study, while limited in generalizability due to its exploratory design, offers valuable insights on enhancing empathy within the healthcare system. It argues that clinical empathy should be viewed as a professional competency that requires institutional commitment and culturally sensitive cultivation. To this end, healthcare policy should focus on structural improvements that foster empathetic care, like reducing provider workload and ensuring patient privacy. Empathy training should be integrated into medical, nursing, and midwifery curricula, emphasizing emotional intelligence and real-world role-playing. In clinical practice, ongoing in-service training, peer reflection forums, and mentorship can support empathy by encouraging providers to share challenges and strategies for emotional engagement.

Ultimately, promoting clinical empathy in Zambia’s MHC system requires a coordinated, multilevel response that combines policy reform, educational innovation, and workplace culture change. By highlighting the lived experiences of MHCPs, this study lays the groundwork for future interventions aimed at institutionalizing empathy in practice and calls for continued research that broadens and deepens our understanding of empathy within low-resource, culturally diverse maternal health contexts.

## Supplemental Material

sj-docx-1-hpq-10.1177_13591053251378961 – Supplemental material for Exploring clinical empathy among maternal healthcare providers in Zambia: Does the heart meet the mind? Insights from a qualitative study Supplemental material, sj-docx-1-hpq-10.1177_13591053251378961 for Exploring clinical empathy among maternal healthcare providers in Zambia: Does the heart meet the mind? Insights from a qualitative study by Lena Halawi, Francis Sichimba, Kalunga Cindy Nakazwe and Atika Khalaf in Journal of Health Psychology

sj-docx-2-hpq-10.1177_13591053251378961 – Supplemental material for Exploring clinical empathy among maternal healthcare providers in Zambia: Does the heart meet the mind? Insights from a qualitative study Supplemental material, sj-docx-2-hpq-10.1177_13591053251378961 for Exploring clinical empathy among maternal healthcare providers in Zambia: Does the heart meet the mind? Insights from a qualitative study by Lena Halawi, Francis Sichimba, Kalunga Cindy Nakazwe and Atika Khalaf in Journal of Health Psychology
