# Supplementary material for: Exploring clinical empathy among maternal healthcare providers in Zambia: Does the heart meet the mind? Insights from a qualitative study
Source: J Health Psychol. 2025 Oct 15;31(5):1966–81. doi: 10.1177/13591053251378961 (PMC13031371; doi:10.1177/13591053251378961)
Supplement: sj-docx-1-hpq-10.1177_13591053251378961 – Supplemental material for Exploring clinical empathy among maternal healthcare providers in Zambia: Does the heart meet the mind? Insights from a qualitative study [file sj-docx-1-hpq-10.1177_13591053251378961.docx]

Supplementary material 1. An overview of sub-themes and themes.

| **Categories** | **Themes** |
| --- | --- |
| *Holistic emotional engagement in patient-centered care – The meaning and attributes of empathy* | *The Multifaceted Nature of Empathy in Maternal Healthcare - From Conceptual Understanding to Practical Application* |
| *Empathy is “Not practical enough”- Disconnect between theoretical knowledge and practical application of clinical empathy* |  |
|  |  |
| *Empathy as a Catalyst for Enhanced Healthcare Efficacy and Patient Engagement* | *The Dual Nature of Empathy in Maternal Healthcare - Enhancing Patient Care while Navigating Professional Boundaries* |
| *The Empathy-Professionalism Balance- Navigating Emotional Engagement and Clinical Objectivity* |  |
|  |  |
| *“Navigating Gender-Related Misinterpretations”*  *“Balancing Professionalism and Patient Familiarity”*  *“Age and Educational Background as Barriers to Empathy”*  *“Socioeconomic Factors and Perceptions of Empathy”*  *“The Role of Personal Beliefs and Emotional Resistance”*  *“Motivation and Positive Outcomes of Empathy”*  *“Environmental Influences on Empathy in Maternal Healthcare”* | *Contextual Dynamics of Empathy in Maternal Healthcare- Balancing Challenges and Cultivating a Patient-centered Approach* |
