# Supplementary material for: Exploring clinical empathy among maternal healthcare providers in Zambia: Does the heart meet the mind? Insights from a qualitative study
Source: J Health Psychol. 2025 Oct 15;31(5):1966–81. doi: 10.1177/13591053251378961 (PMC13031371; doi:10.1177/13591053251378961)
Supplement: sj-docx-2-hpq-10.1177_13591053251378961 – Supplemental material for Exploring clinical empathy among maternal healthcare providers in Zambia: Does the heart meet the mind? Insights from a qualitative study [file sj-docx-2-hpq-10.1177_13591053251378961.docx]

Supplementary material 2. Themes and dimensions of clinical empathy as perceived by Zambian maternal healthcare providers.

| **Theme** | **Category** | **Dimension of Clinical Empathy** |
| --- | --- | --- |
| The Multifaceted Nature of Empathy in Maternal Healthcare – From Conceptual Understanding to Practical Application | Holistic Emotional Engagement in Patient-Centered Care – The Meaning and Attributes of Empathy | Conceptual and emotional understanding in care delivery |
|  | “Not Practical Enough” – Disconnect Between Theoretical Knowledge and Practical Application | Practical challenges in applying empathy under real-world conditions |
| The Dual Nature of Empathy in Maternal Healthcare – Enhancing Patient Care while Navigating Professional Boundaries | Empathy as a Catalyst for Enhanced Healthcare Efficacy and Patient Engagement | Empathy's role in improving care outcomes and patient engagement |
|  | The Empathy-Professionalism Balance – Navigating Emotional Engagement and Clinical Objectivity | Maintaining emotional balance and professionalism |
| Contextual Dynamics of Empathy in Maternal Healthcare – Balancing Challenges and Cultivating a Patient-centered Approach | Navigating Gender-Related Misinterpretations | Influence of gender dynamics on empathetic engagement |
|  | Balancing Professionalism and Patient Familiarity | Navigating over-familiarity while maintaining care boundaries |
|  | Age and Educational Background as Barriers to Empathy | Demographic barriers impacting empathetic communication |
|  | Socioeconomic Factors and Perceptions of Empathy | Misinterpretations tied to socioeconomic status |
|  | The Role of Personal Beliefs and Emotional Resistance | Personal biases and emotional constraints in empathetic care |
|  | Motivation and Positive Outcomes of Empathy | Empathy as a motivational factor enhancing outcomes |
|  | Environmental Influences on Empathy in Maternal Healthcare | Infrastructure’s impact on emotional connectivity |
|  | Environmental Influences on Empathy in Maternal Healthcare | Physical space and privacy as facilitators/barriers of empathy |
